# *Anaplasma platys* and *Rickettsia massiliae* in *Rhipicephalus sanguineus* sensu stricto ticks collected on dogs in the Patagonian region of Argentina

**DOI:** 10.1017/S0031182024000933

**Published:** 2024-07

**Authors:** Lara M. I. Maas, Marina Winter, Verónica Herrmann, Sergio D. Abate, Anna Obiegala, Santiago Nava, Patrick S. Sebastian

**Affiliations:** 1 Institute of Animal Hygiene and Veterinary Public Health, University of Leipzig, 04103 Leipzig, Germany; 2 Rio Negro Research and Transfer Center (CONICET-UNRN), National University of Rio Negro, 8500 Viedma, Argentina; 3 Veterinary clinic “La Victoria”, 8536 Valcheta, Argentina; 4 Dairy Chain Research Institute (IdICaL; CONICET-INTA), 2300 Rafaela, Argentina

**Keywords:** companion animals, rickettsiales, South America, tick-borne diseases, ticks

## Abstract

The aim of this study was to examine the presence of tick-borne rickettsial bacteria in *Rhipicephalus sanguineus* sensu stricto ticks collected from dogs in the Patagonian region of Argentina. Fourteen stray dogs from Valcheta, Río Negro province, Argentina were examined for the presence of *R. sanguineus* s.s. ticks. Ninety ticks were collected and identified to species level. DNA was extracted and analysed by conventional PCR assays for the presence of tick-borne bacteria belonging to the genera *Anaplasma*, *Ehrlichia* and *Rickettsia*. Thirty-three tick pools were tested by different PCR assays of which 3 were positive for Anaplasmataceae bacteria. From the 3 Anaplasmataceae positive samples, 2 partial 16S rDNA sequences were generated and belonging to *Anaplasma platys*, the causative agent of canine cyclic thrombocytopenia. Two tick samples were positive in *Rickettsia* specific PCR assays and were identified by phylogenetic analysis as *Rickettsia massiliae*, a member of the spotted fever group rickettsiae. The results of this study demonstrate the molecular detection of 2 rickettsial bacteria in *R. sanguineus* s.s. in a region of Argentina where no data were available so far.

## Graphical abstract

**Figure:**
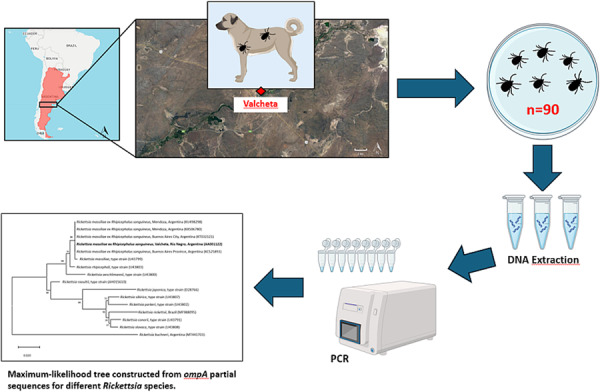

## Introduction

The order Rickettsiales includes obligate intracellular alpha proteobacteria and contains the families of Anaplasmataceae, Midichloriaceae and Rickettsiaceae (Ferla *et al*., 2013). Within this order, several species are of particular importance for veterinary and public health. *Anaplasma platys* and *Ehrlichia canis*, the causative agents of canine cyclic thrombocytopenia and canine monocytic ehrlichiosis, respectively, are amongst the most important bacteria in veterinary medicine (Harrus *et al*., 2012; Atif *et al*., 2021; Diniz and Moura de Aguiar, 2022). *Rickettsia massiliae*, a member of the spotted fever group rickettsiae, is known to be zoonotic human cases are reported (García-García *et al*., 2010; Oteo and Portillo, 2012; Portillo *et al*., 2015).

The main vector of these bacteria is ticks from the *Rhipicephalus sanguineus* group (Bremer *et al*., 2005; Parola *et al*., 2008). In Argentina, 2 different taxa, namely *R. sanguineus* sensu stricto (previously known as *R. sanguineus* sensu lato ‘temperate lineage’) and *R. linnaei* (previously known as *R. sanguineus* sensu lato ‘tropical lineage’) (Nava *et al*., 2017, 2018; Šlapeta *et al*., 2022) are present. *Rhipicephalus linnaei* was found only in the provinces of Formosa and Salta, above 24° southern latitude, while *R. sanguineus* sensu stricto is distributed in almost all regions of Argentina (Nava *et al*., 2012; Cicuttin et al., 2015*a*, 2015*b*; Sebastian *et al*., 2021; Copa *et al*., 2023). Besides *A. platys*, *E. canis* and *R. massiliae*, ticks from the *R. sanguineus* group are involved as potential vector of other pathogenic agents such as *Babesia vogeli*, *Babesia gibsoni* and *Hepatozoon canis* (Dantas-Torres and Figueredo, 2006; Baneth and Allen, 2022).

Knowledge of tick-borne microorganism in the Patagonian region of Argentina is scarce. *Borrelia burgdorferi* sensu lato was detected in *Ixodes* sp. *cf. I. neuquenensis* and *I. sigelos* collected from rodents in the provinces of Chubut, Río Negro and Santa Cruz (Sebastian *et al*., 2016), *Hepatozoon* sp. in *Amblyomma tigrinum* collected on foxes in Santa Cruz province (Millán *et al*., 2018) and ‘*Candidatus* Rickettsia andeanae’ and *Ehrlichia* sp. in *Amblyomma pseudoconcolor* collected on *Chaetophractus villosus* in Río Negro province (Sebastian *et al*., 2022). Further, Winter *et al*. (2024) reported the molecular detection of ‘*Candidatus* Anaplasma boleense’ and two strains of *Ehrlichia* sp. in *A. tigrinum* collected on different hosts and of the vegetation from Rio Negro province, within *Ehrlichia* sp. strain Viedma which may be present in this geographical region associated with the tick species *A. pseudoconcolor* and *A. tigrinum*. However, there are no studies on ticks from the *R. sanguineus* group in the Patagonian region of Argentina.

Therefore, the aim of the study was to investigate the presence of tick-borne rickettsial bacteria with veterinary and public health relevance in *R. sanguineus* s.s. collected on dogs in the Patagonian region from Argentina.

## Material and methods

### Sampling site, tick sampling and identification

Between November 2022 and August 2023, ticks were collected on 14 stray dogs in Valcheta (40°40′47.5″S; 66°09′57.3″W), Río Negro province, Patagonian region, Argentina. This small village is located in a transition area between the ‘Monte’ and ‘Estepa’ phytogeographic areas (Oyarzabal *et al*., 2018).

Ticks were collected manually from the dogs during routine veterinary treatment and investigation. Afterwards, the collected ticks were stored in ethanol (96%), and morphologically determined following Nava *et al*. (2018).

### Molecular detection of tick-borne rickettsial bacteria

Before DNA extraction, ticks were successively washed in hypochlorite (10 vol%), PBS (1X) and distilled water. Afterwards, ticks were pooled in batches of 1–10 specimens, according to tick development stage and individual host. The DNA extraction was carried out with the High Pure PCR Template Preparation Kit (Roche, Germany) according to the manufacturer's instructions. Concentration and purity of the DNA samples were verified *via* Nanodrop measurements. For the detection of rickettsial bacteria, 4 conventional PCR assays were carried out. Shortly, 2 assays detecting different fragments of the 16S rDNA gene of Anaplasmataceae family members were used for the detection of *Anaplasma* spp. and *Ehrlichia* spp. while the genes *gltA* and *ompA* served as targets for the detection of *Rickettsia* species. Used oligonucleotides and assay references are given in Table 1. In all PCR reactions, ultra-pure water was used as negative control while DNA of *Anaplasma marginale* and *Rickettsia parkeri* were chosen as positive control for the detection of the genes for 16S rRNA and *gltA*/*ompA*, respectively.

**Table 1.** Oligonucleotides used for the molecular detection of rickettsial bacteria in DNA samples of *Rhipicephalus sanguineus* sensu stricto ticks collected on dogs from Valcheta, Rio Negro province, Patagonia, Argentina

Primer	Target	Sequence (5′-3′)	References
Ge2-F2	16S rDNA – Anaplasmataceae	GTT AGT GGC AGA CGG GTG AGT	Aguiar *et al*. (2008)
He3		TAT AGG TAC CGT CAT TAT CTT CCC TAT	
EHR16SD	16S rDNA – Anaplasmataceae	GGT ACC YAC AGA AGA AGT CC	Parola *et al*. (2000)
EHR16SR		TAG CAC TCA TCG TTT ACA GC	
CS239	*gltA – Rickettsia* sp.	GCT CTT CTC ATC CTA TGG CTA TTA T	Labruna *et al*. (2004)
CS1069		CAG GGT CTT CGT GCA TTT CTT	
Rr190.70p	*ompA –* spotted fever group (SFG) rickettsiae	ATG GCG AAT ATT TCT CCA AAA	Regnery *et al*. (1991)
Rr190.602p		AGT GCA GCA TTC GCT CCC CCT	

### Data analysis

Positive PCR amplicons were purified using the High Pure PCR Product Purification Kit (Roche, Germany) and sent to INTA Castelar (Genomics Unit, Buenos Aires, Argentina) for sequencing. Obtained partial sequences were edited using BioEdit Sequence Alignment Editor (Hall, 1999) with optional manual edition, aligned with the program Clustal W (Thompson *et al*., 1994), and compared with sequences deposited in GenBank. Phylogenetic analyses for the *gltA* and *ompA* partial sequences were performed with maximum-likelihood (ML) methods by using the program Mega X (Kumar *et al*., 2018). Best-fitting substitution models were determined with the Akaike Information Criterion using the ML model test implemented in MEGA X. Support for the topologies was tested by bootstrapping over 1000 replications, and gaps were excluded from the comparisons.

## Results and discussion

**Figure 1.**
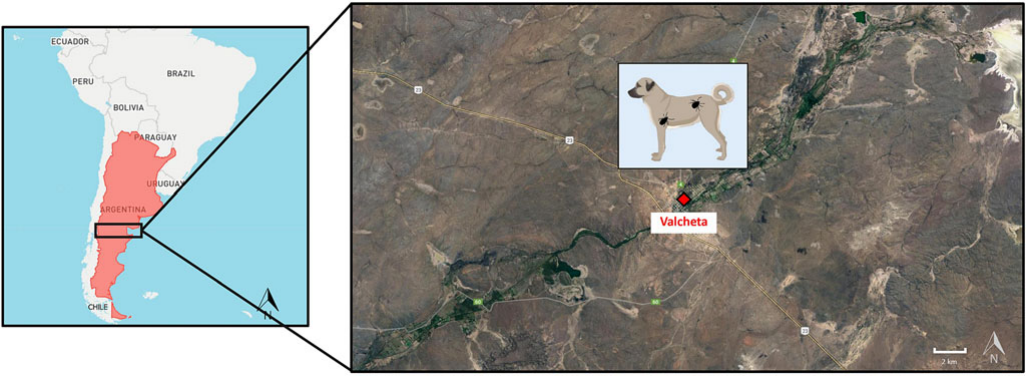
Map of Rio Negro province, Patagonia, Argentina showing the sample locality (Valcheta: 40°40′47.5′′S; 66°09′57.3′′W) of the *Rhipicephalus sanguineus* sensu stricto ticks used for the detection of tick-borne rickettsial bacteria. The map was created with QGIS version Prizren 3.34.0.

A total of 90 specimens of *R. sanguineus* s.s. ticks (3 larvae, 72 nymphs, 6 females and 9 males) were collected on 14 dogs from Valcheta, Río Negro province (see Fig. 1, Table 2). Additionally, a co-infestation of *R. sanguineus* s.s. ticks with 1 female of *A. tigrinum* was observed (this tick was not included in the rickettsial bacterial detection assay). Both tick species are known to infest dogs and were also previously identified in the Patagonian region of Argentina (Nava *et al*., 2017).

**Table 2.** *Rhipicephalus sanguineus* sensu stricto collected on dogs in Río Negro province, Patagonia, Argentina with results of the molecular detection of tick-borne rickettsial bacteria

			No. of *Rhipicephalus sanguineus*				Positive PCR samples (A: Anaplasmataceae; R: *Rickettsia* sp.)			
Dog ID	Locality	Collection date	L	N	F	M	L	N	F	M
VH4	Valcheta	07.12.2022		9	1	3			R^a^	
VH6	Valcheta	12.12.2022			1					
VH7	Valcheta	14.12.2022				1				
VH8	Valcheta	15.12.2022	2	10			A	A		
VH9	Valcheta	20.12.2022				1				
VH10	Valcheta	27.12.2022		18						
VH12	Valcheta	02.01.2023		2						
VH13	Valcheta	02.01.2023				2				
VH15	Valcheta	07.02.2023	1	7	3				R^b^	
VH16	Valcheta	07.02.2023				1				
VH17	Valcheta	10.02.2023		12						
VH18	Valcheta	10.02.2023		8				A		
VH19	Valcheta	14.02.2023		6		1				
VH20	Valcheta	04.03.2023			1					
Total			3	72	6	9	1	2	2	0

L., larva; N., nymph; F., female; M., male.

a positive sample in the *gltA* PCR assay

b positive sample in the *gltA* and *ompA* PCR assays

Thirty-three tick pools were tested by the different PCR assays of which 3 were positive in the 16S rDNA PCR (Parola *et al*., 2000) for Anaplasmataceae bacteria (1 pool of larvae and 2 pools of nymphs), 2 in the *gltA* PCR (2 pools of females) and 1 in the *ompA* PCR (1 pool of females). From the three Anaplasmataceae positive samples, 2 partial 16S rDNA sequences belonging to *A. platys* could be generated: (I) a 273 bp fragment (GenBank acc. no. PP838794) from a pool of 10 nymphs of *R. sanguineus* s.s. and (II) a 301 bp fragment (GenBank acc. no. PP838795) from a pool of 8 *R. sanguineus* s.s. nymphs. Unfortunately, no sequence could be generated from the larval pool. The both obtained sequences were 100% identical to each other (273 bp/273 bp) and with partial sequences of *A. platys* strains detected in Argentina (GenBank acc. no. JX261979, KC525894, KF826282, KF826284) and other parts of the world (e.g. GenBank acc. no. KP717550, KP903296, KT359590, OP164858, OQ132527). *Anaplasma platys* is known as the causative agent of canine infectious cyclic thrombocytopenia (Diniz and Moura de Aguiar, 2022) and was firstly detected in *R. sanguineus* ticks from Northeastern Argentina in 2011 (Oscherov *et al*., 2011). Eiras *et al*. (2013) detected *A. platys* in dogs with suspected rickettsial disease in Buenos Aires province, Argentina. This rickettsial bacterium was also found in dogs from Buenos Aires City (Cicuttin *et al*., 2014), Salta (Dias Cordeiro *et al*., 2020), Córdoba and Santa Fe provinces (Mascarelli *et al*., 2016). Further, Cicuttin *et al*. (2015*a*, 2015*b*) showed the presence of *A. platys* in different *R. sanguineus* populations that belonged to the species *R. linnaei* in northern Argentina. In accordance with the results of the current and previous studies, it can be stated that both species – *R. linnaei* and *R. sanguineus* s.s. – could be potential vector of *A. platys* in Argentina. Laatamna *et al*. (2022) also detected *A. platys* in *R. sanguineus* s.s. ticks collected on dogs from Algeria, which is consistent with the results of the presented study. The vectorial competence of *R. sanguineus* s.s. for *A. platys* was demonstrated under laboratory conditions by Snellgrove *et al*. (2020).

**Figure 2.**
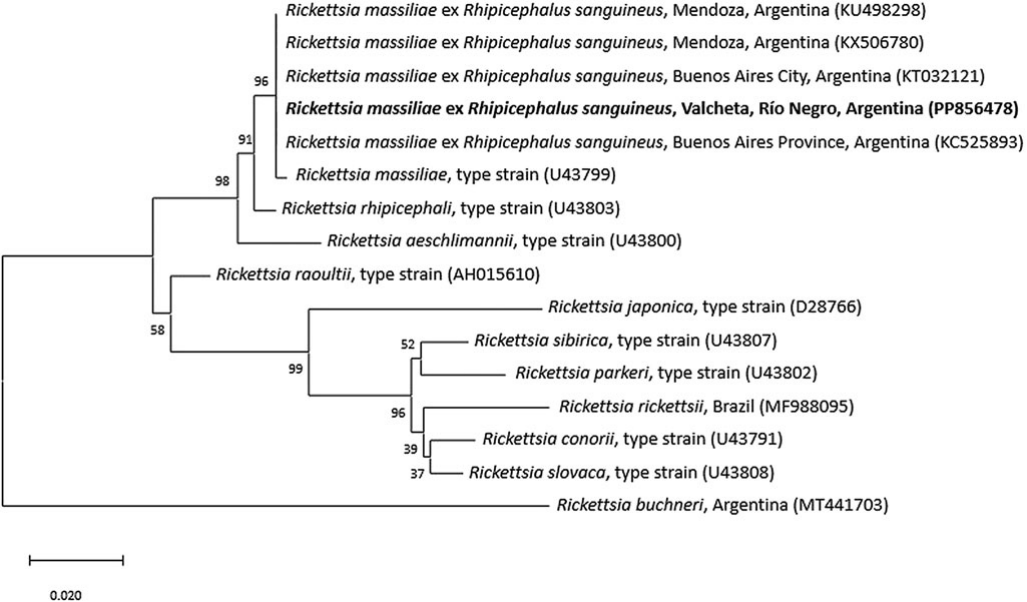
Maximum-likelihood tree constructed from *ompA* partial sequences for different *Rickettsia* species. The partial generated in this study is written in bold letters. Numbers represents bootstrap support generated from 1.000. Genbank accession numbers are given in brackets. The alignment length was 432 bp and the General Time Reversible (GTR) substitution model with Gamma distribution and invariant sites was used.

Two partial sequences of the *gltA* gene from *Rickettsia* spp. were generated from female tick pools collected on different dogs. Both sequences (GenBank acc. no. PP856476, PP856477) showed sequence identities from over 99.24% to different sequences of *R. massiliae*, including some strains detected in Argentina. Further, an *ompA* partial sequence (GenBank acc. no. PP856478) from one of these *gltA* positive pools also could be generated and the BLAST comparison confirmed the identification of the sample as *R. massiliae*. This could be strengthened by the result of the phylogenetic analysis of the sequence (see Fig. 2).

Within the Maximum-likelihood tree constructed from *ompA* partial sequences for different *Rickettsia* species, the sequence generated in this study forms a clade with other strains of *R. massiliae* detected in Argentina and the type strain, which clearly separates (bootstrap value 96) from *Rickettsia rhipicephali* as the next related species. *Rickettsia massiliae* was previously detected in Argentina in canine blood samples and *R. sanguineus* ticks from Buenos Aires City (Cicuttin *et al*., 2004), Buenos Aires province (Cicuttin *et al*., 2014) and Mendoza province (Monje *et al*., 2016). This spotted fever group *Rickettsia* was described for the first time in 1993 (Beati and Raoult, 1993), and it is associated with ticks of the genus *Rhipicephalus.* Further, *R. massiliae* is related to infections in humans, although reports of human cases are rare (Portillo *et al*., 2015). So far, there is one confirmed case of disease in humans caused by *R. massiliae* (García-García *et al*., 2010) reported for Argentina. This association has public health relevance because *R. sanguineus* ticks are known to infest humans (Guglielmone *et al*., 2021).

Within this study we present the molecular detection of 2 rickettsial bacteria associated to *R. sanguineus* s.s. in a region of Argentina where no data about this topic were available so far. To our best knowledge the findings of *A. platys* and *R. massiliae* in *R. sanguineus* represent to most austral reports of these pathogens.

## Data Availability

Partial sequences of the detected rickettsial agents are available on the NCBI GenBank database.
